# Genome-wide transcriptome profiling uncovers differential miRNAs and lncRNAs in ovaries of Hu sheep at different developmental stages

**DOI:** 10.1038/s41598-021-85245-y

**Published:** 2021-03-12

**Authors:** Samina Shabbir, Prerona Boruah, Lingli Xie, Muhammad Fakhar-e-Alam Kulyar, Mohsin Nawaz, Salsabeel Yousuf, Tianyi Liu, Farhat Jabeen, Xiangyang Miao

**Affiliations:** 1 State Key Laboratory of Animal Nutrition, Institute of Animal Sciences, Chinese Academy of Agricultural Sciences, Beijing, 100193 China; 2 Biomass Energy Technology Research Centre, Key Laboratory of Development and Application of Rural Renewable Energy (Ministry of Agriculture and Rural Affairs),Biogas Institute of Ministry of Agriculture and Rural Affairs, Section 4-13, Chinese Academy of Agricultural Sciences, Beijing, China; 3 Shanghai Veterinary Research Institute, Shanghai, Chinese Academy of Agricultural Sciences, Beijing, China; 4 College of Veterinary Medicine, Huazhong Agricultural University, Wuhan, 430070 China; 5 Key Laboratory of Genetics and Germplasm Innovation of Tropical Special Forest Trees and Ornamental Plants, Ministry of Education, College of Forestry and College of Tropical Crops, Hainan University, Haikou, 570228 China; 6 Government College University, Faisalabad, Pakistan

**Keywords:** Biological techniques, Computational biology and bioinformatics, Developmental biology, Genetics

## Abstract

Ovary development is an important determinant of the procreative capacity of female animals. Here, we performed genome-wide sequencing of long non-coding RNAs (lncRNAs) and mRNAs on ovaries of 1, 3 and 8 months old Hu sheep to assess their expression profiles and roles in ovarian development. We identified 37,309 lncRNAs, 45,404 messenger RNAs (mRNAs) and 330 novel micro RNAs (miRNAs) from the transcriptomic analysis. Six thousand, seven hundred and sixteen (6716) mRNAs and 1972 lncRNAs were significantly and differentially expressed in ovaries of 1 month and 3 months old Hu sheep (H1 vs H3). These mRNAs and target genes of lncRNAs were primarily enriched in the TGF-β and PI3K-Akt signalling pathways which are closely associated with ovarian follicular development and steroid hormone biosynthesis regulation. We identified MSTRG.162061.1, MSTRG.222844.7, MSTRG.335777.1, MSTRG.334059.16, MSTRG.188947.6 and MSTRG.24344.3 as vital genes in ovary development by regulating CTNNB1, CCNA2, CDK2, CDC20, CDK1 and EGFR expressions. A total of 2903 mRNAs and 636 lncRNAs were differentially expressed in 3 and 8 months old ovaries of Hu sheep (H3 vs H8); and were predominantly enriched in PI3K-Akt, progesterone-mediated oocyte maturation, estrogen metabolism, ovulation from the ovarian follicle and oogenesis pathways. These lncRNAs were also found to regulate FGF7, PRLR, PTK2, AMH and INHBA expressions during follicular development. Our result indicates the identified genes participate in the development of the final stages of follicles and ovary development in Hu sheep.

## Introduction

Sheep is one of the main livestock in China, which serves as an important source of meat and fur-related products^1^. Improved living standards have partly created a huge increase in the demand for high-quality sheep products. However, current production seldom meets domestic demand across the country^2^. One of the key goals of sheep breeding is high reproductive performance since it plays a major role in production efficiency. Conversely, low fertility is one of the major limiting factors for the growth of the sheep industry. Traditional selection has been used to increase litter size due to low reproductive heritability and sex-limiting nature of local breeds. Nevertheless, candidate genes linked to ovulation and multiplets can lead to genetic improvement through gene and marker-assisted selection (MAS) in sheep^3^. Thus, improving sheep breeding capability and increasing output of mutton and wool products can successfully fill the ever-growing gap in supply and demand while guaranteeing quality of mutton. There are several sheep species globally but only a few of them have high fecundity, while the vast majority produce only single lamb with few multi-born occurrences^4^. Hu is one of China's most important sheep breeds reputed for its early maturity, four seasons of estrus and multiple lambs per birth^5^. In animal reproduction and breeding, the ovaries are the reproductive organs of female mammals. Therefore, it is important to understand their gene expression and regulation. This will provide a basis for further ovarian research at the molecular level to uncover their regulatory mechanisms.

Transcriptomic studies in sheep ovaries offer contemporary insights into breed fertility and make an important contribution to the field of developmental biology^4, 6^. Previously, the majority of transcriptomic studies centered on microarray analyses and were used as an effective way to understand biological pathways at the molecular level^7^. However, the advancement of molecular biology, sequencing and bioinformatics technologies offers a platform for the measurement of large-scale gene expression patterns using high-throughput RNA-sequencing (RNA-seq)^8^.

The RNA-seq have been recently employed to identify differentially expressed genes and novel transcripts in cattle^9^, cows^10^, goats^11^ and pigs^12^. RNA-seq efficacy has also been demonstrated in mammalian reproductive tissues, such as pig gonad^13^, bovine blastocyst^14^, bovine granulosa cell^15^; goat ovary^16^; sheep oocyte and granulosa cell; and sheep ovary^17^. A novel technology of high sequence RNA-seq^18^ in which sequences are singularly traced to the genome source and are computed to procure the density and number of respective RNAs from already known exons^19^ has been employed to analyze genome-wide mRNAs in several species including yeasts, ruminants, and humans^20^.

Non-coding long-chain RNA (LncRNA) and micro-RNA (miRNA) affect ovarian growth, cell proliferation, ageing and apoptosis^6^. LncRNA and miRNAs are co-integrated and regulate all aspects of ovarian tissue development. Even though LncRNA does not encode proteins, it has been shown to have a transcriptional and post-transcriptional effect on gene functions as it is commonly involved in different physiological processes^21^. LncRNA and miRNAs play an important part in the regulation of ovary maturation, ovarian cells development, and hormone secretion^22^. Furthermore, the integration of LncRNA and miRNAs allows a thorough study of their regulatory mechanisms in ovarian growth at the molecular level^6^.

Ovarian tissues of the high-fertility Hu sheep breed at different developmental stages were harvested, their LncRNAs and miRNAs were sequenced to identify and predict differently expressed genes (DEGs) at the various time periods. Gene ontology (GO) and Kyoto Encyclopedia of Genes and Genomes (KEGG) were also used for functional enrichment analyses, LncRNAs and miRNAs involved in their expression levels and the genes associated with Hu sheep ovary development were identified. We further studied ovarian gene expression patterns and their enrichment pathways in Hu sheep to gain a better understanding of their regulatory roles in folliculogenesis.

## Results

### Reads mapping to the Hu sheep transcriptome and quality control

The raw reads ranged from 93,652,970 (H3O2) to 110,137,398 (H8O3) representing 14.05 and 16.52 Gb, respectively (Table 1). After a quality check, the adapters and low-quality reads were eliminated from the data. The cleaned data ranged from 84,794,180 (H3O2) to 99,138,982 (H8O3) reads (Table 1). The sequence reads were mapped to the sheep reference genome using TopHat and implemented in Bowtie^23^. The number of uniquely mapped reads spanned from 79.54% (H3O4) to 89.16% (H1O1) and were mapped to the sheep reference genome (Table 1). Furthermore, the GC contents ranged from 44.14% (H8O3) to 50.61% (H3O4) (Table 1). Approximately > 86% clean reads were successfully mapped on to the sheep reference genome (maximum of two mismatches). The high genome coverage of our RNA-Seq data showed that the samples collected were of high quality.

**Table 1:** Summary of reads of Hu sheep transcriptome.

Sample	Number of all reads	Number of bases (in Gb)	Number of mapped reads	% mapped reads	Number of uniquely mapped reads	% uniquely mapped reads	GC (%)	Q30 (%)
H1O1	101,573,406	15.24	93,452,236	92.00	90,565,997	89.16	44.42	94.85
H1O2	99,943,620	14.99	90,234,408	90.29	86,858,329	86.91	45.70	93.30
H1O3	109,737,692	16.46	97,741,027	89.07	94,687,726	86.29	46.16	92.86
H3O2	93,652,970	14.05	84,794,180	90.54	76,905,319	82.12	49.37	93.43
H3O3	101,226,522	15.18	90,445,904	89.35	82,257,380	81.26	49.52	93.79
H3O4	102,358,914	15.35	88,602,145	86.56	81,416,284	79.54	50.61	93.48
H8O1	99,549,070	14.93	91,578,596	91.99	86,481,421	86.87	45.78	93.40
H8O2	102,949,620	15.44	93,260,211	90.59	90,178,208	87.59	46.22	93.62
H8O3	110,137,398	16.52	99,138,982	90.01	97,211,757	88.26	44.14	93.33

H1O1–H1O3 refers to 3 ovaries of Hu sheep at 1 month old; H3O2–H3O4 refers to 3 ovaries of Hu sheep at 3 months old and H8O1–H8O3 refers to 3 ovaries of Hu sheep at 8 months old.

### Identification of differentially expressed genes in the Hu sheep ovaries at different stages of development

**Figure 1:**
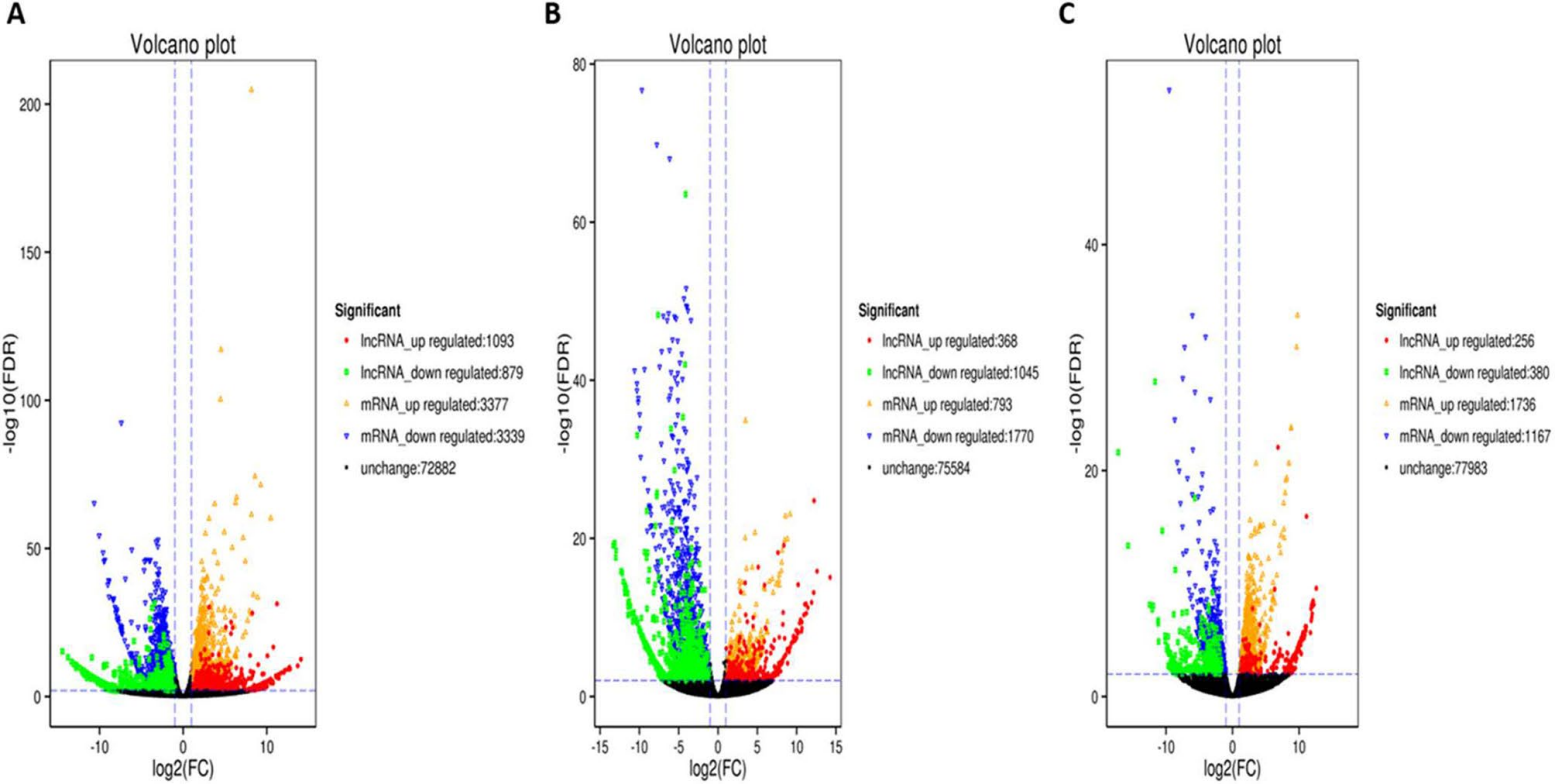
Volcano plots showing the distribution of differentially expressed long non-coding RNAs (lncRNAs) and messenger RNAs (mRNAs) in ovarian tissues in Hu sheep. (**A**) H1 vs H3, (**B**) H1 vs H8 and (**C**) H3 vs H8.

Through the comparative analysis of the ovarian tissues in two groups (H1 vs H3) and (H3 vs H8), log_2_FC > 1.5 and FDR < 0.05 were used as screening criteria. A total of 36,580 lncRNAs and 44,990 messenger RNAs (mRNAs) including novel coding regions were detected in the ovarian tissues of H1 vs H3 (Fig. 1). However, 6716 mRNAs differed in expression, out of these, 3377 were up-regulated, while 3339 were down-regulated (Fig. 1). Similarly, 1972 lncRNAs were differentially expressed, 1093 were up-regulated, and 879 were down-regulated in H1 vs H3. Conversely, 72,882 genes did not differ in their expression in the H1 vs H3 group (Fig. 1A total of 1280 mRNAs and 1413 lncRNAs were differentially expressed in H1 vs H8. In comparison with H3 and H8, a total of 36,549 lncRNAs were identified, out of these, 636 were differentially expressed (256 were up-regulated and 380 were down-regulated) (Fig. 1). Also, 2903 out of 44,973 mRNAs were differentially expressed in the H3 vs H8 group. These consisted of 1736 up-regulated and 1167 down-regulated mRNAs (Fig. 1). There was no variation in the expression of 77,983 genes detected. Individually, DEGs identified among the nine samples (H1O1, H1O2, H1O3, H3O2, H3O3, H3O5, H8O1, H8O2 and H8O3) positively correlated with each group/sample (r = 0.85–0.99) (Supplementary Figure S1a,b). Comparatively, a total of 118 differentially expressed unique genes were identified simultaneously among the three pairwise groups (Supplementary Figure S1c; Supplementary Table S2). These consistently detected DEGs comprised 72 genes with known functions, whereas 46 are novel genes (Supplementary Table S2). These unique DEGs may be screened further as potential candidate biomarkers for marker-assisted breeding applications.

### Differentially expressed coding and non-coding genes and novel miRNA prediction

The secondary structures of the inverted repeats were predicted, and novel micro RNAs (miRNAs) regulating mRNAs (detected in this study) were identified using the RNA fold. We detected 238 novel miRNAs, and 244 long non-coding RNAs (lncRNAs) with significant differences in H1 vs H3, and H1 vs H8 ovarian groups respectively (Table 2). An average of 42,899, 42,899 and 42,885 miRNAs were detected among the three biological replicates of 1-month-old (H1), 3 months old (H3) and 8 months old (H8), respectively (results not shown). Out of these, 101, 114 and 116 miRNAs of known functions with a significant difference were found in H1 vs H3, H1 vs H8 and H3 vs H8, respectively (Table 2). Also, among the significant miRNAs, 486, 606 and 548 were novel with unknown functions in H1 vs H3, H1 vs H8 and H3 vs H8, respectively (Table 2).

**Table 2:** Number of differentially expressed coding and non-coding genes identified from H1 vs H3, H1 vs H8 and H3 vs H8.

Differentially expressed	H1 vs H3	H1 vs H8	H3 vs H8
mRNAs	6716	2563	2903
lncRNAs	1972	1413	636
Known miRNAs	101	114	116
Novel miRNAs	486	606	548

H1 vs H3 = H1O1–H1O3 and H3O2–H3O4 represent 3 ovaries from 1 and 3 months old, respectively; H1 vs H8 = H3O2–H3O5 and H8O1–H8O3 represent 3 ovaries from 3 and 8 months old, respectively, and H3 vs H8 = H3O2–H3O5 and H8O1–H8O3 represent 3 ovaries from 3 and 8 months old, respectively.

### GO and KEGG pathway enrichment analyses

Results of the GO analysis for H1 vs H3 and H3 vs H8 following GO terms; cellular components, biological processes and molecular functions were markedly associated with ovary development and hormone production (Supplementary Figure S2). The 3 top biological processes consisted of progesterone metabolic process, ovarian follicular development and germ cell development (Supplementary Figure S2a). In terms of molecular function, H1 vs H3 were mainly enriched in growth factor binding, insulin-like growth factor receptor binding, estrogen receptor binding, and cyclin-dependent protein activity. The cellular components identified were significantly enriched in high-density lipoprotein particle, voltage-gated calcium channel complex and RNA polymerase transcription factor complex (Supplementary Figure S2a).

Similarly, differentially expressed mRNAs in H3 vs H8 were mainly enriched in the estrogen metabolic process, ovarian follicle development from the ovarian fold, meiosis I, oocyte development, oogenesis, regulation of insulin-like growth factor receptor signalling pathway, transforming growth factor-beta production and ovulation cycle. In terms of molecular function, DEGs were enriched in transforming growth factor beta binding, insulin-like growth factor binding, estradiol 17 beta dehydrogenase activity and calcium dependent ATPase activity. Also, GO terms associated with vesicle membrane and laminin complex were identified as cellular components (Supplementary Figure S2b). GO analysis showed that, the differential mRNAs were primarily enriched in oocyte development.

**Figure 2:**
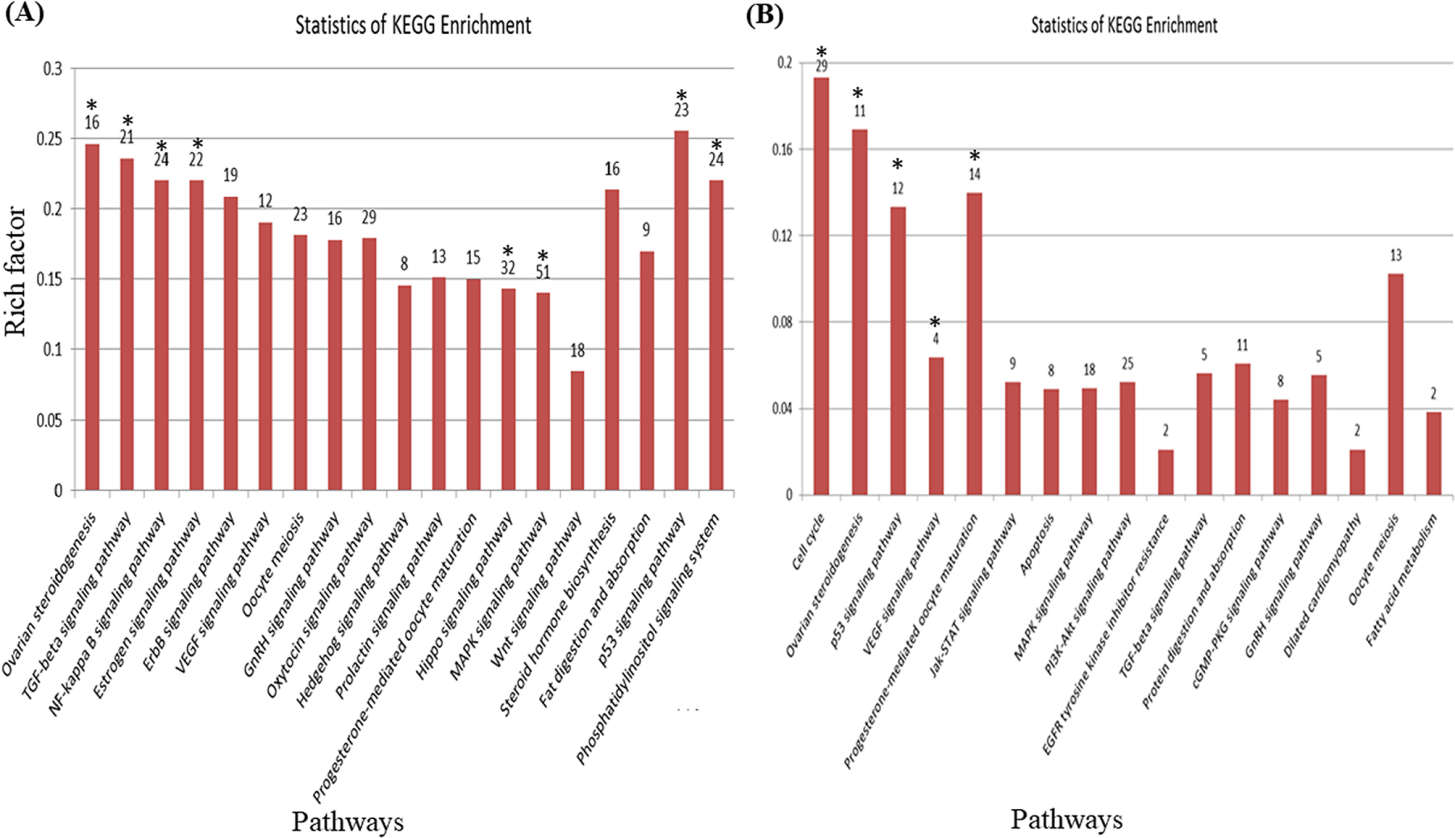
KEGG pathway analysis of differentially expressed mRNAs in ovaries of Hu sheep at (**A**) H1 vs H3 and (**B**) H3 vs H8. H1, H3 and H8 represent ovaries sampled at 1, 3 and 8 months old, respectively. The x-axis represents the name of the pathway, and the y-axis represents the rich factor corresponding to each pathway (the number of differential mRNA enriched in each pathway/the number of all genes enriched in this pathway in the background gene concentration). The numbers on top of each bar represent the number of differential mRNA enriched in each pathway. Bars with * indicates those highly significant enriched with *p* value ≤ 0.001.

KEGG offers a comprehensive database resource for interpretation of fully sequenced genomes through biological signal pathways^24^. Among the differentially expressed mRNAs in H1 vs H3, ovarian steroidogenesis, TGF-β signalling, estrogen signalling, Rap1 signalling pathway, Hippo signalling pathway, MAPK signalling, NF kappa B signalling, p53 signalling, and phosphatidylinositol signalling pathways were identified (Fig. 2A). The enrichment analysis of KEGG pathway showed that the differentially expressed mRNAs in ovarian tissues of H1 vs H3 Hu sheep participate in multiple pathways associated with ovarian follicles development. The development of various parts of the ovary is characterized by a series of phenomena that affect sexual maturity. This is important for studying functions of associated mRNAs in the regulation of ovary development.

Conversely, cell cycle, ovarian steroidogenesis, p53 signalling pathway, progesterone-mediated oocyte maturation and EGF signalling pathways were identified as the key pathways in H3 vs H8 group (Fig. 2B). These pathways align with the GO terms implicated in oocyte development. From the results of KEGG pathway enrichment analyses, we deduce that differential lncRNAs may affect these pathways by regulating mRNAs in ovary development.

### Interaction networks between lncRNAs and target genes associated with ovary development

**Figure 3:**
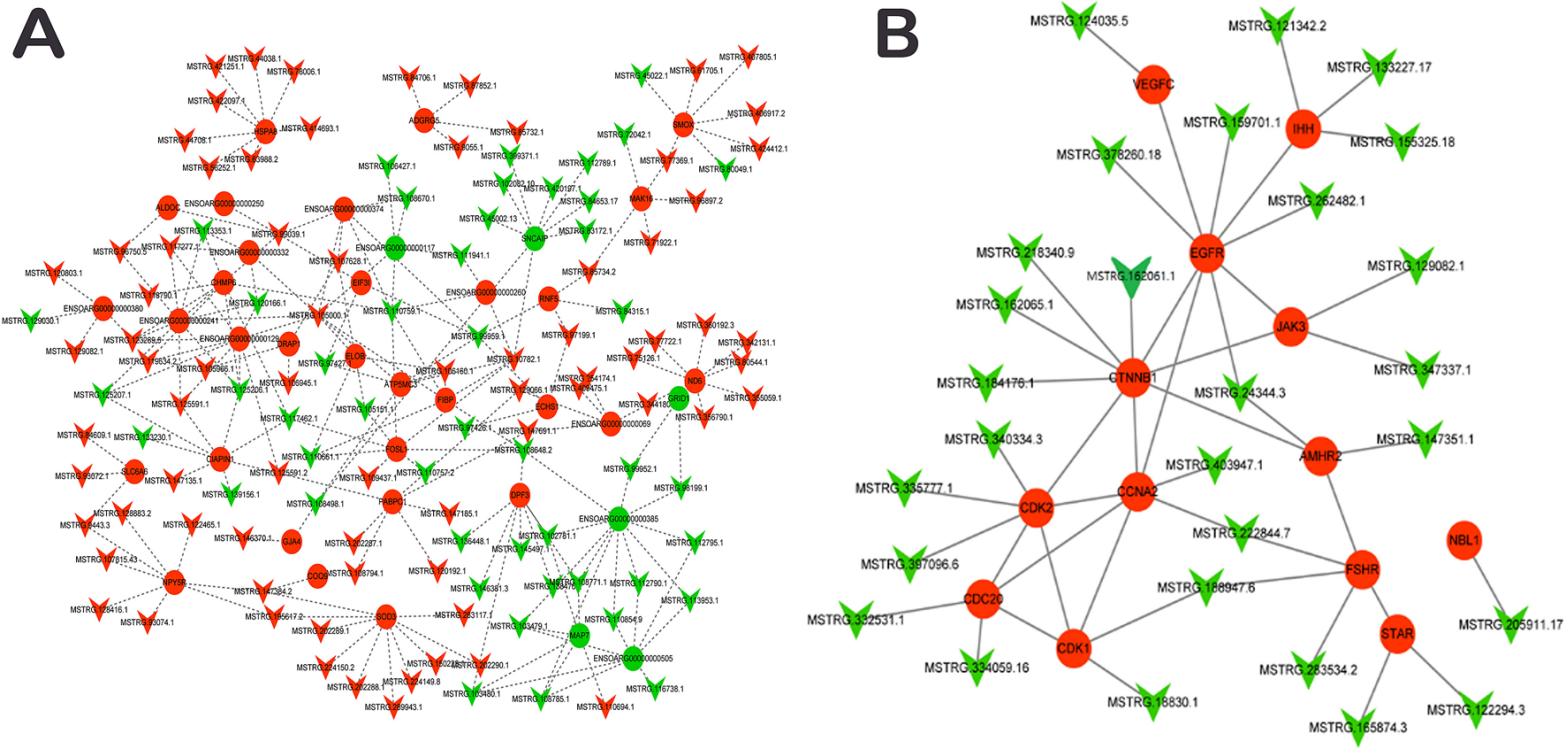
Co-expression network of the differentially expressed lncRNAs and mRNAs in ovary tissues in H1 vs H3 (**A**) H3 vs H8 (**B**). Triangle and circle nodes represent lncRNA and mRNAs, respectively. The nodes with red colour represent up-regulation, whereas those with green represents down-regulation. The dotted line represents the target relationship between two nodes.

**Figure 4:**
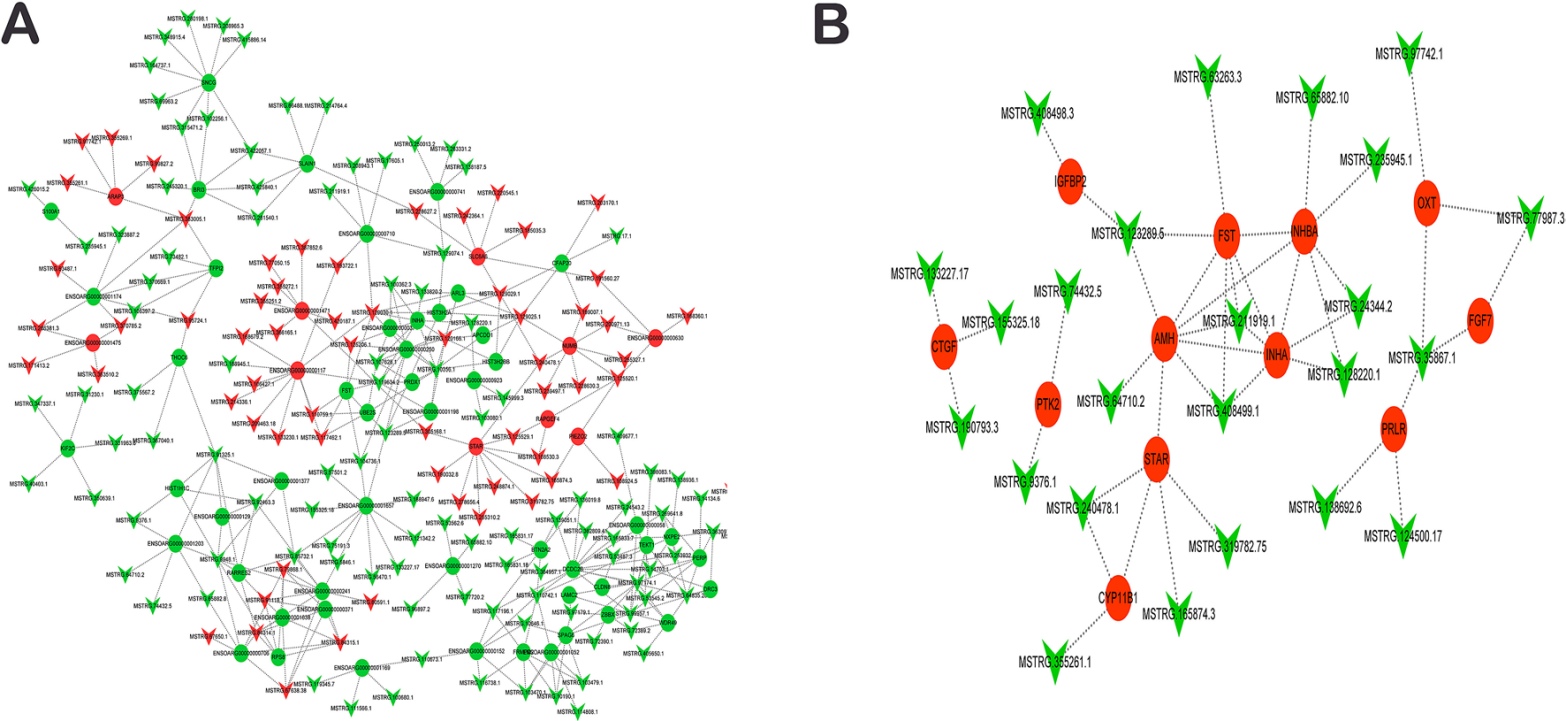
Co-expression network of lncRNAs and differentially expressed mRNAs related to ovarian development in H1 vs H3 (**A**) H3 vs H8 (**B**). Triangle and circle nodes represent lncRNAs and mRNAs, respectively. The nodes with red colour represent up-regulation whiles those with green colour represent down-regulation. The dotted line represents the target relationship between two nodes.

The potential interaction between lncRNAs and the target genes associated with ovary development was constructed in the Cytoscape software. The *cis* and *trans* regions of lncRNAs were differentially expressed in H1 vs H3 and H3 vs H8 groups. The estimated absolute correlation coefficient values were more than 0.9 with a *p* value less than 0.01 and were subsequently used to study potential roles of the lncRNAs on the mRNAs. The results evidenced that, lncRNA regulates multiple mRNAs (Figs. 3A, 4A). By integrating the results of differential mRNAs and functional annotation of lncRNA target genes, several genes closely related to ovarian development were differentially expressed. Follicle-stimulating hormone receptor (FSHR), catenin (cadherin associated protein), ß1 (CTNNB1), anti-Müellerian hormone type-2 receptor (AMHR2), cyclin A2 (CCNA2), steroidogenic acute regulatory protein (STAR) were significantly enriched in ovaries of H1 vs H3 group (Fig. 3B). These genes were enriched in cAMP signalling, ovarian steroidogenesis, TGF-β signalling, PI3K-Akt signalling, progesterone mediated oocyte maturation and oocyte maturation pathways and involved in vesicle development. We also identified fibroblast growth factors 7 (FGF7), prolactin receptor (PRLR), protein tyrosine kinase 2 (PTK2), anti-Müllerian hormone (AMH), secretion of inhibin A (INHB), and STAR genes in H3 vs H8 (Fig. 4B). These genes are involved in meiosis I process, oocyte development, oogenesis, islet-like growth factor binding, insulin-like growth factor receptor signal and JAK-STAT signal pathways.

### Protein–protein interaction among differentially expressed genes related to ovarian development

**Figure 5:**
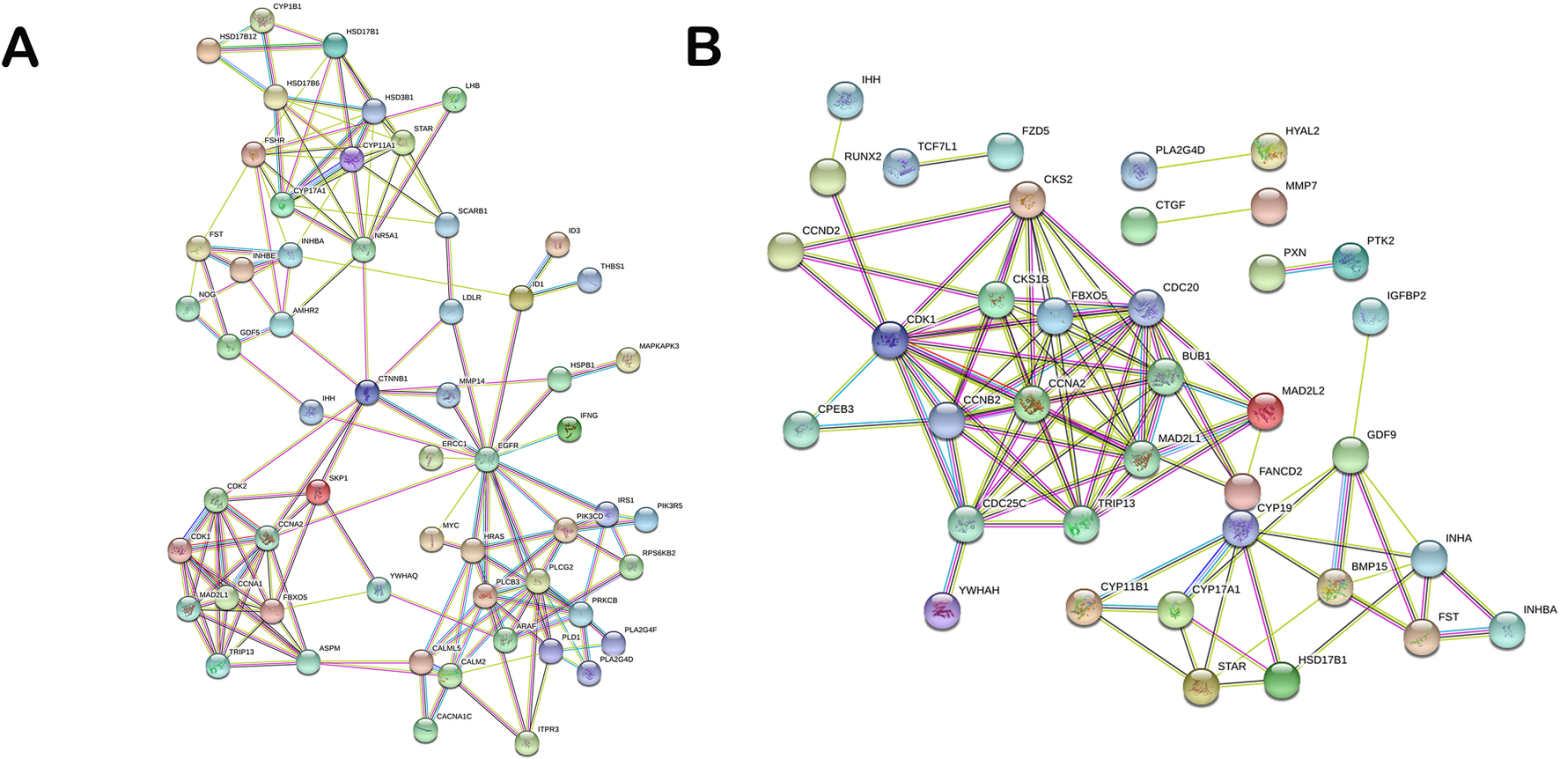
Protein–protein interaction network of differentially expressed messenger RNAs (mRNAs) related to ovary development. (**A**) Probable functional network of mRNAs in H1 vs H3. (**B**) Probable functional network of mRNAs in H3 vs H8. H1, H3 and H8 represent 1, 3 and 8 months old ovaries, respectively.

Through GO and KEGG pathway enrichment analyses, related differential mRNAs were selected for protein–protein interaction network analysis. The network was constructed using the STRING database^25^. This gives an idea on the probable functional network of mRNAs that regulate ovarian growth and development. Key genes such as FSHR, CDK2, EGFR, CCNA2, CYP11A1, GDF5, STAR were detected to be hub-genes (genes with high correlation in candidate modules, thus, those with high connectivity ranked at top 10%) in the protein–protein interaction for H1 vs H3 (Fig. 5A). At the same time, STAR, INHB, INHA, CDC20 and GDF9 were the key hub-genes in H3 vs H8 protein–protein interaction (Fig. 5B). These genes have previously been reported to play significant roles in regulating ovary growth and development^3, 26^. Most of the hub-genes identified were down-regulated in the ovaries sampled.

### Validation of RNA-seq data by quantitative real time PCR

**Figure 6:**
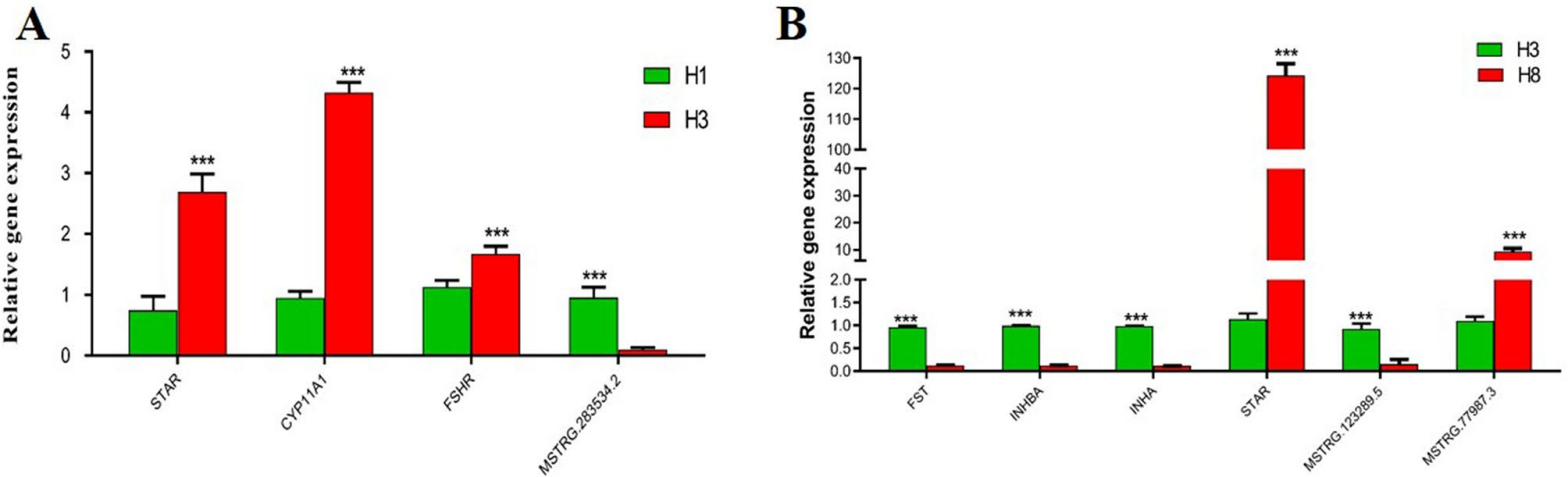
Relative expression of 10 selected differentially expressed genes. (**A**) Expression of 4 genes in H1 vs H8. (**B**) Expression of 6 genes in H3 vs H8. H1, H3 and H8 represent 1, 3 and 8 months old ovaries, respectively. The error bars represent the standard deviation. Bars with an asterisk (**) indicate significant difference by t test at 5% probability.

The results of quantitative real time PCR (qRT-PCR) showed STAR, CYP11A1 and FSHR were significantly up-regulated in 3-month-old ovaries (H3), while MSTRG.283534.2 was significantly down-regulated (Fig. 6A). In the 8-month-old ovaries (H8), FST, INHB, INHA and MSTRG.123289.5 were significantly down-regulated, while STAR and MSTRG.77987.3 were significantly up-regulated (Fig. 6B). These results are in consonance with our RNA-seq results. These genes had high expression either as up- or down-regulated in RNA-seq results; hence, providing a clue for their potential functional validation in our subsequent experiments.

## Discussion

### Genome-wide identification of mRNAs, miRNAs and lncRNAs in ovaries of Hu sheep

Ovarian development in ruminants has been directly linked to lncRNA and miRNA regulatory mechanisms as it is implicated in ovarian disease prevention and senescence, cell proliferation and apoptosis^16^. The co-regulatory role of lncRNAs and miRNAs in ovarian tissue development has received limited research attention^4, 27, 28^. Hitherto, few studies have explored miRNA expression profiles in mammalian ovary development^4^. Numerous lncRNAs and mRNAs are encoded in mammals, but the function of most lncRNAs are yet to be fully studied^29^. Many studies have linked lncRNA disorders to the reproduction, including germ cell formation, early embryo implantation and ovulation^30, 31^ and reproductive hormone regulation^33^.

We identified a total of 101, 114 and 116 differentially expressed miRNAs with known functions in the H1 vs H3, H1 vs H8 and H3 vs H8 in a pairwise fashion, respectively (Table 2) in ovaries of Hu sheep. The key miRNAs identified include oar-let seven families (a–d, f, g and i). The oar-let seven family is one of the first discovered miRNA groups, and its family members are highly conserved in sequences across animal species^34^. The oar-let seven family were predominantly downregulated in H1 vs H3 compared to H1 vs H8 and H3 and H8 groups (up-regulated). These changes observed at the miRNA expression level could control different pathways. Also, miRNAs have been estimated to regulate more than one-third of protein-encoding mRNAs^28^. Studies have shown that many non-coding RNAs such as miRNAs are involved in the regulation of ovarian development, influencing the reproductive ability of animals, and are closely related to ovarian tissue-related diseases^35, 36^. The 5 top most differentially expressed miRNAs identified in the present study included oar-miR-148a, oar-miR-21, oar-let-7b, oar-let-7a and oar-let-7b and were also highly expressed in the ovaries of goats in earlier studies^37, 38^, pigs^39^, mice^40^, cattle^41^ and other animal species Li, et al.^42^. These miRNAs indicate their involvement in ovary development by modulating the mRNAs as an important regulator of reproductive processes^43^. However, their fundamental roles in modulating mRNAs need further functional validation.

The transforming growth factor β (TGF-β) signalling pathway has multi-functions that regulate cell growth, differentiation, apoptosis, movement and invasion, extracellular matrix production, angiogenesis and immune response^44^. More significantly, the TGF-β signalling pathway has multiple functions in mammalian ovary growth in sheep. The differential genes enriched in this TGF-β signalling pathway include AMHR2 and NBL1. AMHR2 belongs to the type II receptor family of TGF-β-related proteins—a serine/threonine kinase that is found in granules and follicle membrane cells. The first step in transformation of follicle development is the activation of primordial follicles. AMH can inhibit the activation of follicles in mouse ovaries and function through the type 2 receptor (AMHR2)^45, 46^. AMHR2 was down-regulated in the ovary of 3-months old Hu sheep and regulated by MSTRG.147351.1 and MSTRG.24344.3, which can effectively reduce the inhibitory effect of AMH on follicular development and promote activation and growth of embryonic follicles.

The gonadal hormone regulates transcription, the inhibitory effect of BMP2 and BMP4 on granulosa cells during follicular development. Thus, NBL1 may affect the development of Hu sheep ovary through the TGF-β signalling pathway^47^. The PI3K-Akt signalling pathway plays an important role in follicular development and participates in the interaction between oocytes and surrounding cumulus cells^48^. In this study, EGFR, CDK2, JAK3, and VEGFC were differentially enriched in the PI3K-Akt signalling pathway. The development and differentiation of ovarian follicles are characterized by significant changes in the expression of aromatase (CYP19A1). However, its down-regulation requires effective signal transduction through EGFR^49^. Hormone-induced steroid production and primordial follicle growth are essential to EGFR signalling in cumulus cells as a key factor in oocytes development^50^.

The different lncRNAs, MSTRG.244344.3, MSTRG.262482.1 and MSTRG.378260.18 identified in the 1 and 3 months old ovaries of Hu sheep may be involved in regulating the development of ovary follicles by up-regulating EGFR. The protein encoded by CDK2 is the catalytic subunit of the cyclin-dependent protein kinase complex, which regulates cell cycle development. Similarly, cyclic ovarian activity is key to reproductive success and profound changes in tissue composition. Its function requires exquisite spatiotemporal coordinated proliferation, apoptosis and differentiation of many different cell types within follicles, corpora lutea and ovarian stroma^30^. Sugiura et al.^51^ reported that blocking CDK2 activity can lead to cyclin B1 accumulation and a failure to induce meiosis. Thus, CDK2 participates in meiotic maturation of mammalian oocytes, promoting the first oocyte transition to the second meiosis. MSTRG.335777.1, MSTRG.340334.3, MSTRG. 397096.6 may have a *trans* effect on CDK2 and affect follicle maturation. JAK signalling regulates the formation of primitive follicles in mice and participates in the proliferation of granulosa cells, and its inhibition (JAK3) reduces the proliferation of granulocytes. Inhibition of JAK3 expression can also occasion failure in primordial follicles formation^52^. Previous studies have shown that VEGFC and VEGF-D can co-integrate with VEGFA to stimulate early events in the angiogenesis of primate ovulatory follicles. However, the function of VEGFC in mammalian ovarian development is still unknown. Thus, lncRNAs may affect the PI3K-Akt pathway by regulating the expression of EGFR, CDK2, JAK3, and VEGFC in ovarian development.

### Differentially expressed mRNAs, miRNAs and lncRNAs in ovaries of Hu Sheep

In this study, FSHR was up-regulated, and FSH activates FSH receptor (FSHR) in granulosa cells to induce follicular differentiation, growth, and estradiol production^53^. In domestic animals, FSHR is only expressed in the GC of the ovary and plays a key role in apoptosis, atresia, follicle maturation, GC proliferation and differentiation, and ovulation^54^. The mRNA level of FSHR in the ovarian follicle is similar to that of sheep in an earlier study by Goyal et al. (2017). Three lncRNAs, MSTRG.188947.6, MSTRG.222844.7, MSTRG.283534.2 were predicted to regulate the expression of FSHR, which may be associated with follicle development^55^. We screened and predicted MSTRG.122294.3 and MSTRG.165874.3 as target genes. The *trans* effect regulates the expression of STAR; an indispensable component in the acute regulatory phase. It mediates the transfer of cholesterol from the outer membrane to the inner membrane of the mitochondria to form the first type of sterol, which regulates the synthesis of sex hormones. This indicates that differential lncRNAs may mediate ovarian hormone synthesis by influencing STAR. CCNA2 has different roles in mitosis and meiosis in female animals as CCNA2 and CDK coordinate mitosis and promote oocytes to enter meiosis, regulating the transition of meiosis I to meiosis II^56^.

The GO enrichment analysis revealed several significantly enriched biological processes, molecular functions and cellular components which are closely associated with ovarian tissue development and hormone production (Fig. 2; Supplementary Figure S2). Viral carcinogenesis biosynthetic pathway was the common enriched pathway in H1 vs H3; while Rap1, Ras, PI3k-Akt, oxytocin, neurotrophin, MAPK signalling, endocytosis and cell adhesion molecules (CAMs) signalling pathways were among the preponderantly enriched pathways in H3 vs H8 group (Supplementary Figure S2b). The Rap1 signalling pathway regulates cell adhesion, cell–cell junction formation and cell polarity, cycling between inactive GDP-bound and active GTP-bound conformations^57, 58^. Rap1 acts as a switch during cellular signalling transduction regulated by binding to either guanosine triphosphate (GTP) or guanosine diphosphate (GDP)^59^. The Pl3k-Akt signalling pathway, endocytosis and cell adhesion molecules (CAMs) were identified in the H1 vs H8 group; In contrast, Rap1 signalling, PI3k-Akt signalling, olfactory transduction, cell adhesion and CAMs pathways were predominantly enriched in the H3 vs H8 group. These enriched pathways are favorable for cellular processes in the ovaries and potentially contribute to the high folliculogenesis in the Hu sheep (Supplementary Figure S2a,b).

Our KEGG pathway analysis was consistent with the GO analysis. The miRNA and lncRNA target genes were significantly enriched in Rap1 signalling, PI3k-Akt signalling, olfactory transduction, cell adhesion and CAMs biosynthesis pathways. The PI3k-Akt signalling pathway induces transcription of target genes to mediate angiogenesis, cell invasion, metastasis, proliferation and apoptosis^60^. We detected six DEGs of WD repeat domain (WDR; 16, 63, 74, 76, 77 and 83) which have been functionally linked to enzyme binding^61^. The WDR70 gene was reported as a candidate gene for fertility traits in Chinese and Nordic Holsteins^62^. Recently, Pitt et al.^63^ identified a BTA20 region associated with several cattle loci for feed intake, milk traits, mastitis, mutability and reproduction. This further indicates the functional role of WDR in ovarian development of the Hu sheep.

The calcium, estrogen, insulin, MAPK and PI3K-Akt-signaling pathways (Figs. 3, 4) are associated with gonadal development, ovarian steroidogenesis, oocyte maturation and steroid hormone biosynthesis^64^. These pathways may play important roles in the regulation of follicle development in the Hu sheep. As a negative regulator of stress-activated MAP kinase (MAPK) signalling pathways, calcium pathway down-regulates inositol 1,4,5-trisphosphate receptor-dependent calcium signalling. It promotes cardiomyocyte hypertrophy via activation of the calcineurin/NFAT signalling pathway^65^.

DEGs from several pathways shown in the interaction network obtained from the STRING database emphasized that ovary development which is a major determinant of prolificacy in sheep, may be regulated by multiple genes from different signalling pathways. The unique miRNAs and lncRNAs identified in this study may serve as potential selection signatures for identifying new candidate genes, and novel candidate regions involved in ovary development of Hu sheep. Our study provides valuable evidence for further molecular research on ovary development in the Hu sheep breed. However, further studies are needed to investigate the functional roles of some of the selected DEGs, especially novel genes identified in the Hu sheep ovaries.

## Conclusion

We employed the next-generation high-throughput sequencing technology and bioinformatics tools to identify lncRNAs and mRNAs of Hu sheep ovaries at different development stages. We found that miRNAs and lncRNAs were differentially expressed in the three comparison groups at the varying developmental stages. Six thousand seven hundred and sixteen (6716) differentially expressed mRNAs (3377 up-regulated, 3339 down-regulated, including unknown genes) and 1972 differentially expressed lncRNAs (1093 up-regulated, 879 down-regulated) were identified in the ovaries of 1 and 3 months old Hu sheep. The GO annotation and KEGG enrichment analysis of *cis* and *trans* target genes and differential mRNAs revealed the lncRNA target genes and differential mRNAs might be involved in ovarian follicle development, steroid hormone-mediated signalling pathways, steroid hormone biosynthesis, gonadotropin response, and insulin-like growth factor receptor binding. The target genes of differential mRNAs and lncRNAs in the ovaries of 1 and 3 months old sheep were mainly enriched in the TGF-β and PI3K-Akt signalling pathways, ovarian follicle development and steroid hormone biosynthesis. The target genes of differential mRNAs and lncRNAs in the ovaries of 3 and 8 months old sheep were primarily enriched in PI3K-Akt, ovarian steroid production, progesterone-mediated oocyte maturation, estrogen metabolism, ovarian follicle ovulation and oogenesis pathways. Candidate lncRNAs involved in the development of Hu sheep ovarian follicles and key target genes identified may require further studies by knockout, overexpression, and RNAi to authenticate lncRNAs-specific functions in ovarian development.

## Materials and methods

### Animal and ovary sample collection

BB female Hu sheep were raised under normal conditions at Linqing Animal Husbandry Co., Ltd (Shandong, China). The sheep were divided into three groups-1 month old (H1) (with weights 12.7 kg, 12.38 kg and 12.75 kg), 3 months old, (16.65 kg, 17.52 kg and 18.3 kg) and 8 months old (40.64 kg, 39.86 kg and 40 kg). Three biological replicates were used for each group: H1(H1O1, H1O2 and H1O3), H3 (H3O2, H3O3 and H3O5) and H8 (H8O1, H8O2 and H8O3). The animals were slaughtered by exsanguinations according to a protocol (GB/T 17236-2008) approved by State Administration for Market Regulation and Standardization Administration, following deep anesthesia with tiletamine/zolazepam (Zoletil 50 Vet, Virbac, France) (tiletamine 50 mg/ml and zolazepam 50 mg/ml), at a dose of 0.1 mg/kg of body weight, administered by intramuscular injection. Horizontal bloodletting was performed immediately after fainting, and the bloodletting from stabbing didn’t exceed 30 s. The length of the knife is about 5 cm. The bleeding was not less than 5 min. After mechanical depilation, the floating hair and dirt was washed in the clean water pool. The tissues were collected and frozen in liquid nitrogen and stored immediately at – 80 °C for further RNA extraction. All the experimental procedures were carried out according to authorization granted by the Chinese Ministry of Agriculture^66^.

### RNA extraction, sequencing libraries and RNA-Seq

The Hu sheep ovaries were harvested and total RNA was extracted from the ovaries using the TRIZOL RNA extraction kits (Invitrogen, Carlsbad, CA). RNA concentration and purity were checked using Nano Drop 2000 spectrophotometer (Thermo Fisher Scientific, Wilmington, DE). The integrity of the RNA was validated using the RNA Nano 6000 Assay Kit (Agilent Technologies, CA, USA). A total of 1.5 μg of RNA was used per sample as the input material to remove rRNAs. This was done using Ribo-Zero rRNA removal kit (Epicentre, Madison, WI, USA). The Sequencing libraries for six samples (HPgroup, n = 3; LP group, n = 3) were generated by NEBNextR UltraTM Directional RNA Library Prep Kit for IlluminaR (NEB, USA). The sequences for each of the samples were labelled with index codes. Fragments of 150-2 bps were selected by purifying the library fragments using AMPure XP beads (Beckman Coulter, Beverly, USA). Three (3) μlUSEREnzyme (NEB, USA) was subsequently used alongside size-selected, adaptor-ligated cDNAs at 37 °C for 15 min prior to polymerase chain reaction (PCR). The PCR was then run using Phusion High-Fidelity DNA polymerase, Universal PCR primers and index (X) Primer. PCR products were finally purified (AMPure XPsystem, Beckman Coulter, Beverly, MA, USA) and quality of the library was evaluated using Agilent Bioanalyzer 2100 through quantitative real-time PCR (qRT-PCR). The previously index-coded samples were aggregated on acBot Cluster Generation System using TruSeq PE Cluster Kitv3-cBot-HS (Illumia) following the manufacturer’s instructions. Library preparations were sequenced using Illumina Hiseq platform after cluster generation, and paired-end reads were generated. The purity and size of the library were assessed by the Agilent 2100 system (Agilent, California). The transcript sequences of ovaries used for the study have been deposited in the Sequence Read Archive (SRA) repository of the National Center for Biotechnology Information (NCBI) (SRA accession number: PRJNA638028).

### Read mapping and prediction of lncRNAs and miRNAs

RNA-Seq reads from each of the samples were matched by TopHat to the Oar_4.0-sheep reference genome using the default settings^23^. LncRNAs were identified following the procedure by^67^. Briefly, transcripts were blasted to known mRNAs, and other types of non-coding RNAs (including pseudogenes, pre-microRNA, tRNA) were eliminated. Then, the transcripts with single exon and length < 200 bp were excluded from further analyses. PLEK^68^, CNCI^69^, CPC^70^ and Pfam^71^ were used to identify the protein-coding potential of the above-obtained transcripts and the intersection of the results were taken as the final results. Finally, in order to identify known lncRNAs, BLASTN tool was used to align lncRNA candidates to ALDB (a domestic animal extended non-coding RNA database), a database with a focus on domestic animal lncRNAs with the following settings, identity = 100%, mismatch = 0, E value < 1e−10 and gap_opening = 0^72^.

The clean reads were aligned to the miRNA precursor/mature miRNA of *Ovis aries* in miRBase 21.0 (http://www.mirbase.org/ftp.shtml) to identify the sequences and counts of miRNAs observed in the samples. The characteristics of hairpin structures of miRNA precursors were used to predict novel miRNAs. Milarepa v0.2 (http://sourceforge.net/projects/mireap/) was used to analyze the unannotated reads.

For gene expression analysis, only uniquely mapped reads were used. The *DESeq* package in R software^73^ was used to classify substantially and differentially expressed genes (DEGs) according to the stringent significance test for the automated gene expression profiling earlier described by Anders and Hubers^74^. False discovery rate (FDR) was adopted for the error rate adjustment in multiple significance tests^75^. If the log_2_fold change (FC) was > 1.5 and the FDR was < 0.05, the miRNAs and lncRNAs were considered to be differentially expressed.

### Differential gene expression analysis and functional annotation

A correlation analysis was also performed between miRNAs and mRNAs. If the correlation between any two pair is > 0.9 between candidate miRNAs and the putative targets, the gene was considered as the correct target of the miRNA. These links between novel miRNAs and mRNAs were further illustrated by the functional networks of the miRNA-mRNA pairs. Functional enrichment analyses were performed using the Gene Ontology (GO)^76^ and KEGG^24^ databases. In the GO analysis, genes and gene products covered by three domains are described in a controlled vocabulary: molecular function, cell component and biological process. In this study, GO categories were applied to understand the potential roles of DEGs and miRNA target genes^76^. In the KEGG database, significant pathways were identified for the predicted target genes at the adjusted p  ≤ 0.05 set as the significant threshold.

### Protein–protein interaction analysis

The path-act-network analysis was conducted to identify the interactive network function of pathways enriched with the KEGG-based differential mRNAs, including membrane transportation, metabolism, cell cycles and signal transduction pathways. The STRING database was employed to perform gene-act-network analysis to uncover the network of the differentially expressed mRNAs based on relationships extracted from the database^25^. Networks were developed based on complementary pairs between miRNA and mRNAs, and between miRNA and lncRNAs to infer the role of lncRNAs in ovaries of the Hu sheep. miRNAs, lncRNAs and mRNAs were included in the networks. Cytoscape (ver. 2.8) was employed to visualize the miRNA-lncRNA-mRNA networks.

### Quantitative real-time PCR

The quantitative real-time PCR (qRT-PCR) analysis was performed as described previously^6^. Briefly, a total of 1 μg of RNA was reverse transcribed using RT-reagent kits with gDNA Eraser (Takara, China) as per the manufacturer’s protocols. Total RNA (0.5 μg) was used to synthesize the first-strand cDNA using PrimerScript RT reagent Kit (TaKaRa Biotech, Dalian, China; code: DRR037A). Each cDNA sample was diluted 10 times in ddH_2_O, and 1 μl of this dilution was used as a template for qRT-PCR. The qRT-PCR reactions were performed in a 10 μl volume containing 5 μl 2 × QuantiFast SYBR Green Master Mix (TIANGEN Biotech, Dalian, China; code: FP204), 1 μl cDNA, and 0.2 μl of forward and reverse primers (both in 5 μM) and 3.6 μl nuclease-free H_2_O in a Roche HOLD CYCLE LightCycler 480 II ((Roche, Mannheim, Germany). The amplification conditions were 95 °C for 15 min of the initial stage, followed by 40 cycles of 95 °C for 10 s and 60 °C for 30 s. The relative gene expression levels were estimated using the ${2}^{-\Delta \Delta Ct}\mathrm{ method}$^77^. Data obtained were analyzed using GraphPad Prism (vers 7; GraphPad Software, San Diego California USA, https://www.graphpad.com/scientific-software/prism/). The student t-test (*p* < 0.05) was used for mean comparisons. All results were presented in bar charts with the means and their standard deviation (± SD). Primers used for the qRT-PCR are listed in Supplementary Table S1.

### Ethics statement

All the procedures involving animals were approved by the animal care and use committee at the Institute of Animal Sciences, Chinese Academy of Agricultural Sciences, where the study was conducted. All the experiments were performed in accordance with the relevant guidelines and regulations set by the Ministry of Agriculture of the People’s Republic of China. This study was carried out in compliance with the ARRIVE guidelines.

## Supplementary Information

Supplementary Information 1.

Supplementary Information 2.

## Data Availability

The materials and datasets used and analyzed during the present study are available from the corresponding author upon reasonable request.
