# Supplementary material for: Genome-wide transcriptome profiling uncovers differential miRNAs and lncRNAs in ovaries of Hu sheep at different developmental stages
Source: Sci Rep. 2021 Mar 12;11:5865. doi: 10.1038/s41598-021-85245-y (PMC7971002; doi:10.1038/s41598-021-85245-y)
Supplement: Supplementary file 1 — Supplementary Information 1. [file 41598_2021_85245_MOESM1_ESM.docx]

Genome-wide transcriptome profiling uncovers differential miRNAs

and lncRNAs in ovaries of Hu sheep at different developmental stages

Samina Shabbir**^#,^** ^1,2^, Prerona Boruah**^#,^** ^3^, Lingli Xie**^#,^** ^1^, Muhammad Fakhar-e-Alam Kulyar^4^, Mohsin Nawaz^5^, Salsabeel Yousuf^1^, Tianyi Liu^1^, Farhat Jabeen^6^, Xiangyang Miao*^1^

| **Gene** | **Forward primer** | **Reverse primer** | **Product length(bp)** | **Annealing Temperature(℃)** |
| --- | --- | --- | --- | --- |
| STAR | CCACACTCTACGAGGAGAT | CAGGTGAGTTTGGTCCTTG | 115 | 60 |
| CYP11A1 | TCCTACCCACAGAGATATGACA | TTCCAGGCTCCTGACTTCTTA | 98 | 60 |
| FSHR | TCCAACCTGCCCAAACTAC | AAGGTTCTGGAAGGCATCA | 84 | 60 |
| MSTRG.283534.2 | AAGGGCACCTGATTATGGTA | ACGCTGTTCTGAGTATCG | 82 | 60 |
| FST | GTCTGTGCCAGTGACAAT | CGGAGTGCTTCACTTCCA | 94 | 60 |
| INHBA | GCAAGGTCAACATTTGCTGTA | TCACAGTAGTTGGCGTGGTA | 106 | 60 |
| INHA | ACCTCGGATGGAGGTTAC | GGATTCCCTTAGATGCAAGC | 83 | 60 |
| MSTRG.123289.5 | TTTCAGCATTAGAGGCACCTAC | GTGAAGTCACACGCCTTATT | 107 | 60 |
| MSTRG.77987.3 | ATGGAGCTGCAAAGAATCG | TAGGTCATGCTGAGTGAGTTAG | 90 | 60 |
| GAPDH | TGACCTTCACTACATGGTCT | ACTTGATGTTGGCAGGAT | 146 | 60 |

**Supplementary Table 1: List of primers used for qPCR experiment**

**
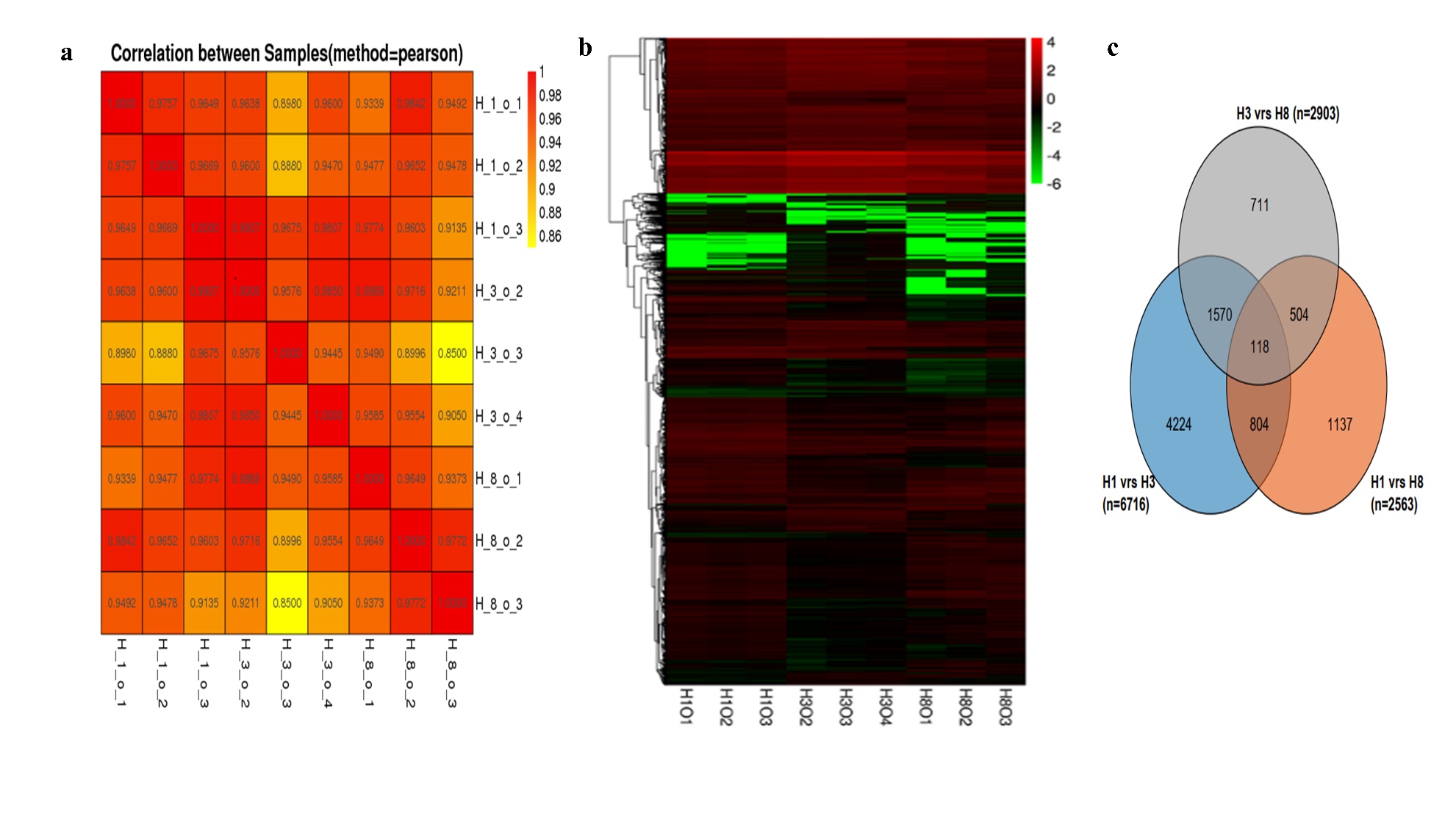
**

Supplementary Figure 1 | Differentially expressed genes among the nine samples used in this study. (a) Correlation among differentially expressed genes identified among the 9 samples used in this study. (b) Heatmap of expression of genes identified among the 8 samples. (c) Venn diagram illustrating the differentially expressed genes among the 3 pairwise groups simultaneously.^1^


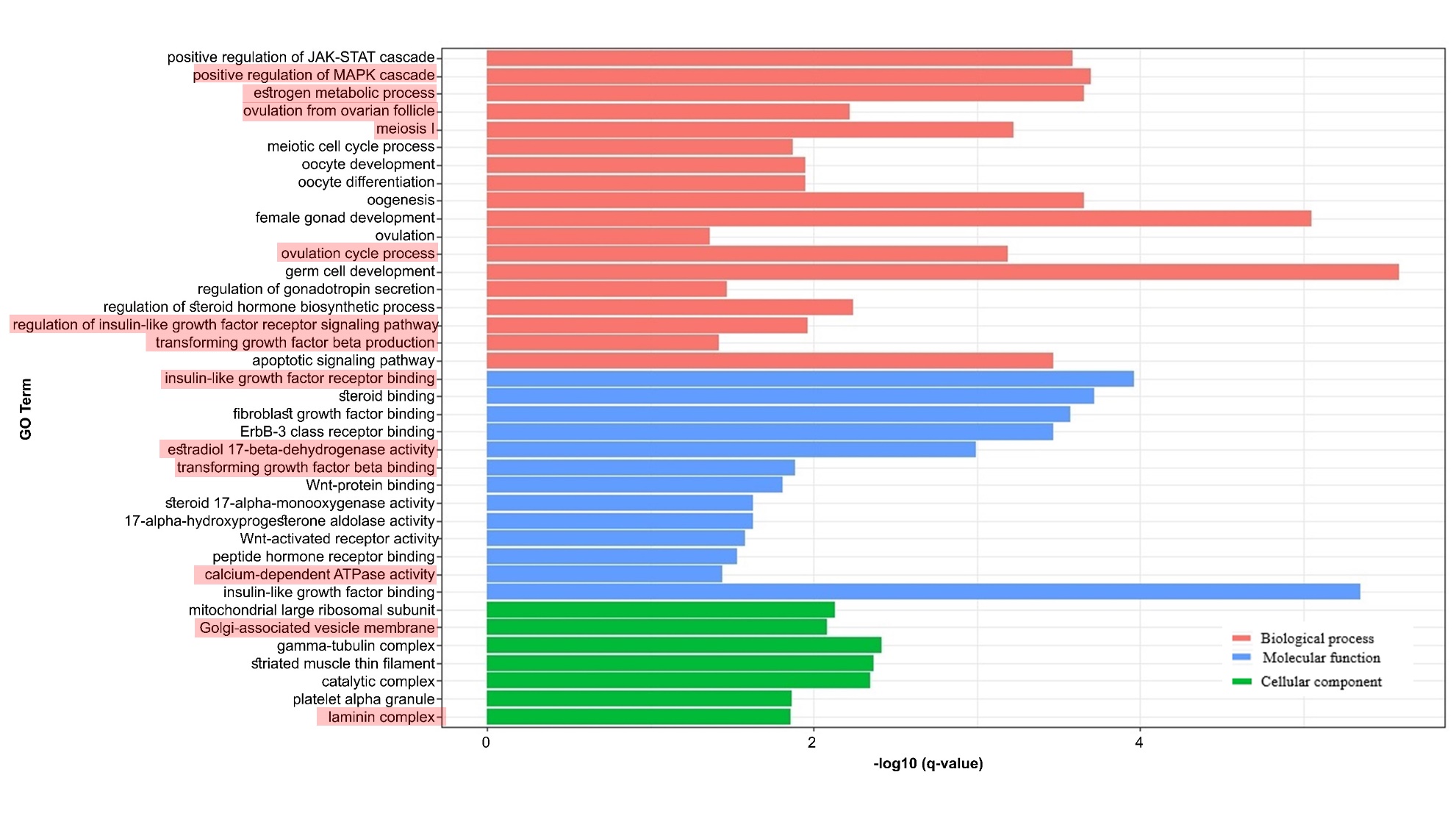


**a**


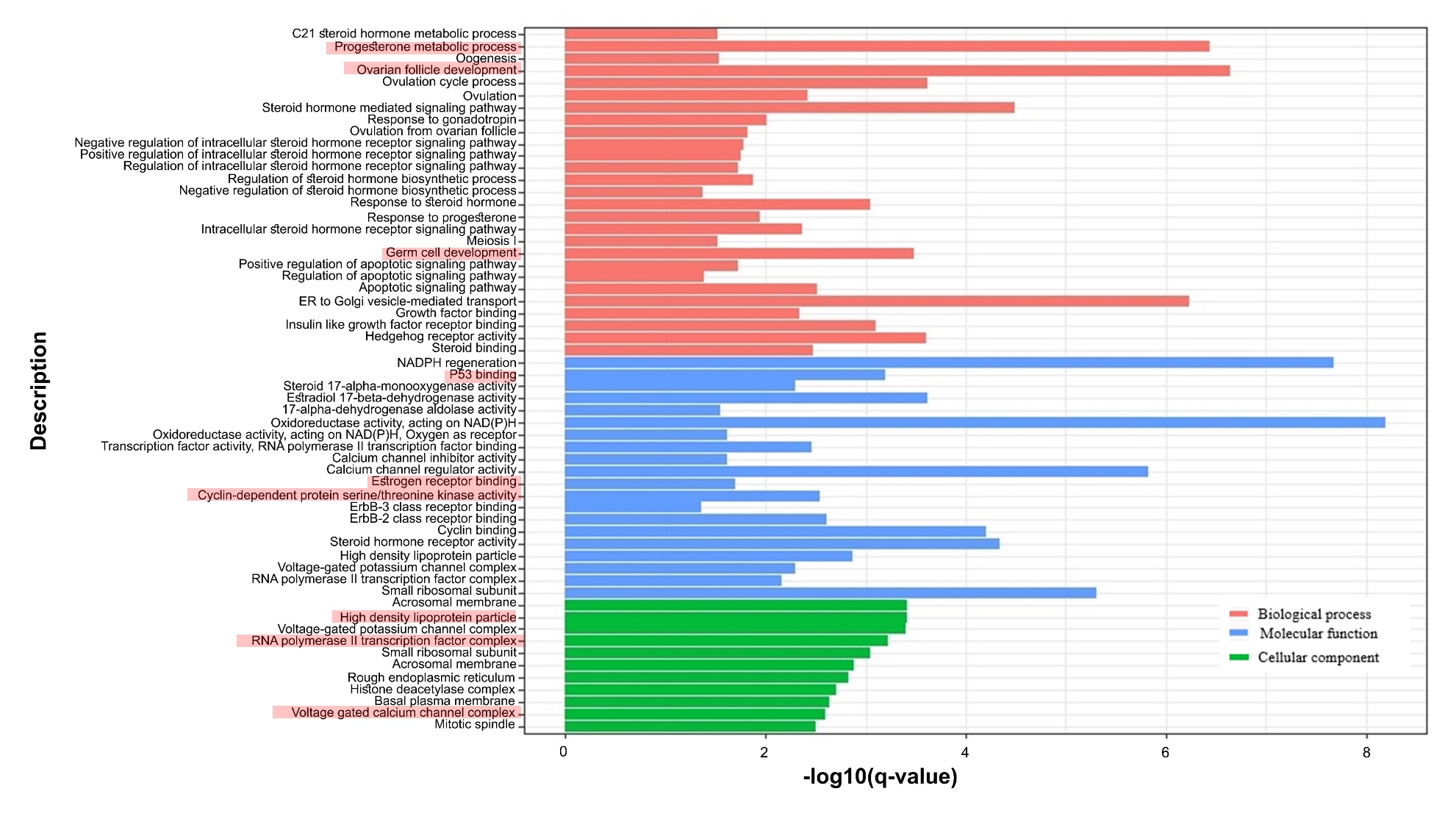


**b**

Supplementary Figure 2 | Functional classification of differentially expressed genes identified from each comparison group based on Gene Ontology (GO). GO analysis was conducted to determine the enrichment of genes in biological process (red bars), cellular components (green bars) and molecular function (blue bars) with P< 0.05. However, those highly enriched with *P*-value ≤ 0.001 have been shaded red. (a) H1 vs H3 (b) H3 vs H8. H1, H3 and H8 represents 1, 3 and 8 month old ovaries from Hu sheep, respectively.^1^

Supplementary references

1 R: A Language and Environment for Statistical Computing v. 3.5.1 (R Foundation for Statistical Computing, Vienna, Austria, 2018).
